# Association of neurofilament light chain with renal function: mechanisms and clinical implications

**DOI:** 10.1186/s13195-022-01134-0

**Published:** 2022-12-16

**Authors:** Rongxiang Tang, Matthew S. Panizzon, Jeremy A. Elman, Nathan A. Gillespie, Richard L. Hauger, Robert A. Rissman, Michael J. Lyons, Michael C. Neale, Chandra A. Reynolds, Carol E. Franz, William S. Kremen, For the Alzheimer’s Disease Neuroimaging Initiative

**Affiliations:** 1 Department of Psychiatry, University of California San Diego, La Jolla, CA 92093 USA; 2 Center for Behavior Genetics of Aging, University of California San Diego, La Jolla, CA 92093 USA; 3 Department of Psychiatry, Virginia Institute for Psychiatric and Behavior Genetics, Virginia Commonwealth University, Richmond, VA 23284 USA; 4 QIMR Berghofer Medical Research Institute, Brisbane, Queensland Australia; 5 Center of Excellence for Stress and Mental Health (CESAMH), VA San Diego Healthcare System, San Diego, CA 92093 USA; 6 Department of Neurosciences, University of California San Diego, CA 92093 La Jolla, USA; 7 Department of Psychological and Brain Sciences, Boston University, Boston, MA 02212 USA; 8 Department of Psychology, University of California Riverside, Riverside, CA 92521 USA

**Keywords:** Neurofilament light chain, Renal function, Neurodegeneration, Blood-based biomarker, Neurodegenerative diseases, Biometrical twin modeling

## Abstract

**Background:**

Blood-based neurofilament light chain (NfL) is a promising biomarker of neurodegeneration across multiple neurodegenerative diseases. However, blood-based NfL is highly associated with renal function in older adults, which leads to the concern that blood-based NfL levels may be influenced by renal function, rather than neurodegeneration alone. Despite growing interest in using blood-based NfL as a biomarker of neurodegeneration in research and clinical practices, whether renal function should always be accounted for in these settings remains unclear. Moreover, the mechanisms underlying this association between blood-based measures of NfL and renal function remain elusive. In this study, we first evaluated the effect of renal function on the associations of plasma NfL with other measures of neurodegeneration. We then examined the extent of genetic and environmental contributions to the association between plasma NfL and renal function.

**Methods:**

In a sample of 393 adults (mean age=75.22 years, range=54–90), we examined the associations of plasma NfL with cerebrospinal fluid (CSF) NfL and brain volumetric measures before and after adjusting for levels of serum creatinine (an index of renal function). In an independent sample of 969 men (mean age=67.57 years, range=61–73) that include monozygotic and dizygotic twin pairs, we replicated the same analyses and leveraged biometrical twin modeling to examine the genetic and environmental influences on the plasma NfL and creatinine association.

**Results:**

Plasma NfL’s associations with cerebrospinal fluid NfL and brain volumetric measures did not meaningfully change after adjusting for creatinine levels. Both plasma NfL and creatinine were significantly heritable (*h*^2^=0.54 and 0.60, respectively). Their phenotypic correlation (*r*=0.38) was moderately explained by shared genetic influences (genetic correlation=0.46) and unique environmental influences (unique environmental correlation=0.27).

**Conclusions:**

Adjusting for renal function is unnecessary when assessing associations between plasma NfL and other measures of neurodegeneration but is necessary if plasma NfL is compared to a cutoff for classifying neurodegeneration-positive versus neurodegeneration-negative individuals. Blood-based measures of NfL and renal function are heritable and share common genetic influences.

## Background

Blood-based biomarkers have garnered increasing attention from clinical and research communities for their low invasiveness and utility in the detection and monitoring of neurodegenerative pathophysiology [1]. As a biomarker of neurodegeneration, the neuron-specific heteropolymer neurofilament light chain (NfL) is an axoskeletal protein that maintains large-caliber myelinated axons and is released into the extracellular space following neuroaxonal damage [2, 3]. Elevated NfL concentrations are detected in both cerebrospinal fluid (CSF) and blood in mild cognitive impairment (MCI), Alzheimer’s disease (AD), frontotemporal dementias, traumatic brain injury, and other neurological conditions [1, 3].

Despite the diagnostic and prognostic potential of blood-based NfL (measured in either plasma or serum) across multiple neurological disorders [2–6], there have been growing calls for caution in interpreting elevated blood-based NfL levels as an indicator of neurodegeneration in older populations without considering common underlying comorbidities that also influence its concentration [5, 7–12]. Specific comorbidities associated with higher blood-based NfL levels include chronic kidney disease, neurological conditions (e.g., stroke), and cardiovascular diseases [10, 11]. Notably, declining renal function, but not increasing number of comorbidities in older adults, has been associated with elevated blood-based NfL levels [13]. Indeed, levels of blood-based NfL are correlated with multiple blood-based measures of renal function (*r*=±0.49–0.56), including serum creatinine, cystatin C, and estimated glomerular filtration rate, in both healthy older adults and those with diabetes [5, 7–10, 13]. Even after controlling for known confounding factors of blood-based NfL such as age and body mass index [7–9, 13], these associations remain. Thus, diminished renal function in older adults appears to be one of the strongest comorbidities contributing to elevated blood-based NfL levels.

Given the growing interest in using blood-based NfL in research and clinical practices [5, 12, 14], as well as the emerging emphasis on considering the impact of comorbidities on blood-based biomarkers of neurodegenerative disorders to avoid potential misdiagnosis [10–12, 15], there is a critical need to evaluate the impact of renal function on blood-based NfL levels with respect to the associations of blood-based NfL with other measures of neurodegeneration unrelated to renal function [1] and investigate the mechanisms underlying this blood-based NfL and renal function association [2]. Addressing these questions would inform best practices in research and clinical settings that use blood-based NfL to index neurodegeneration and enable a better understanding of the nature of these associations.

Because CSF NfL may be a more direct measure of neurodegeneration less influenced by peripheral clearance than blood-based NfL measured in either plasma or serum [16], we first examined whether the association between plasma NfL and CSF NfL changes after controlling for serum creatinine levels (i.e., an index of renal function) in 396 older adults (mean age=75.17 years) (study 1). We also ascertained whether associations between brain volumetric measures, which are alternate indices of neurodegeneration, and plasma NfL are affected by creatinine levels. Any substantial changes in these associations would indicate that renal function is a major factor influencing the accuracy of plasma NfL as a biomarker of neurodegeneration. Next, in a slightly younger sample of 969 community-dwelling twin men (mean age=67.57 years), we replicated the same analyses examining the effect of renal function on plasma NfL’s associations with other measures of neurodegeneration. Finally, we leveraged biometrical twin modeling to determine the extent to which the association between plasma NfL and renal function (i.e., serum creatinine) is explained by shared genetic and environmental influences (study 2).

## Methods

### Participants

#### Study 1

To examine the associations among serum creatinine, CSF NfL, and plasma NfL, we obtained publicly available biomarker data from the Alzheimer’s Disease Neuroimaging Initiative (ADNI) database (adni.loni.usc.edu). The ADNI was launched in 2003 as a public-private partnership, led by principal investigator Michael W. Weiner, MD. The primary goal of ADNI has been to test whether serial magnetic resonance imaging, positron emission tomography, other biological markers, and clinical and neuropsychological assessment can be combined to measure the progression of MCI and early AD. In the current study, we used the ADNI1 dataset. Hereafter, we referred to the dataset as ADNI. Of the 572 participants with usable data for at least two biomarkers, we excluded 176 participants who had a clinical diagnosis of AD and 3 participants with stage 4 chronic kidney disease, resulting in a total of 396 ADNI participants who fell within stage 1 (normal) to stage 3 (moderate) chronic kidney disease (estimated glomerular filtration rate (eGFR) ≥ 30 ml/min/1.73 m^2^) (mean age=75.22 years, SD=6.35; range=54–90) (Table 1). The small set of stage 4 chronic kidney disease participants (*N*=3) was excluded because of their extreme outlying data points.

**Table 1:** Sample characteristics of ADNI participants with available serum creatinine, plasma, and CSF NfL data

Variables	Mean (SD)		
	Everyone (*N* = 393)	Male (*N* = 236)	Female (*N* = 157)
Age	75.22 (6.35)	75.68 (6.39)	74.53 (6.25)
Years of education	15.86 (2.96)	16.41 (2.86)	15.03 (2.92)
Body mass index^+^	26.24 (3.99)	26.45 (3.56)	25.92 (4.56)
Estimated glomerular filtration rate (eGFR)^+^ (mL/min/1.73 m^2^)	69.77 (14.80)	68.79 (14.85)	71.25 (14.65)
Serum creatinine (mg/dL)	0.98 (0.24)	1.08 (0.23)	0.83 (0.17)
Plasma NfL^*^ (ng/L)	38.94 (26.02)	39.17 (25.58)	38.58 (26.75)
CSF NfL^^^ (pg/mL)	1387.13 (1037.74)	1497.93 (1142.21)	1216.67 (828.48)
*Race/ethnicity*			
% White/non-Hispanic	93% (367)	94% (222)	92% (145)
% others	7% (26)	6% (14)	8% (12)
*Mild cognitive impairment*			
% yes	51% (200)	56% (132)	43% (68)
% no	49% (193)	44% (104)	57% (89)

Non-residualized scores reported for biomarkers

^*^Seven missing (2 males, 5 females) (*N* = 386)

^+^Two missing (1 male, 1 female) (*N* = 391)

^^^Ninety-six missing (56 males, 40 females) (*N* = 297)

#### Study 2

To replicate the analyses from study 1 in an independent sample, as well as examine the genetic and environmental contributions to the NfL-renal function association, we examined 974 community-dwelling male-male twins from wave 3 of the Vietnam Era Twin Study of Aging (VETSA) with available biomarker data. VETSA is a multisite national longitudinal study of aging and risk for AD beginning in middle age [17]. All participants served in the US military some time between 1965 and 1975. Approximately 80% report no combat exposure. Moreover, they are similar to American men in their age range with respect to health, education, and lifestyle characteristics [18]. All traveled to the University of California San Diego or Boston University for the VETSA project. Informed consent was obtained from all participants and institutional review boards at both sites approved all protocols.

Of the 974 participants with usable data, 5 who met the criteria for stage 4 (severe) or stage 5 (end stage) chronic kidney disease (eGFR < 30 ml/min/1.73 m^2^) were excluded, resulting in a total of 969 participants who fell within stage 1 (normal) to stage 3 (moderate) chronic kidney disease (mean age=67.57 years, SD=2.52; range=61–73). The small set of stage 4 and 5 chronic kidney disease participants (*N*=5) were excluded because of their extreme outlying data points. There were 218 monozygotic (MZ) pairs, 151 dizygotic (DZ) pairs, and 231 unpaired individuals (i.e., participants whose co-twin had no available data) (Table 2).

**Table 2:** Sample characteristics of VETSA participants (*N* = 969)

Variables	Mean (SD)
Age	67.57 (2.52)
Years of education	13.98 (2.09)
Body mass index^*^	29.93 (5.20)
Estimated glomerular filtration rate (eGFR)^+^ (mL/min/1.73 m^2^)	75.43 (13.79)
Serum creatinine^+^ (mg/dL)	1.04 (0.21)
Plasma NfL (ng/L)	13.18 (6.88)
*Race/ethnicity*	
% White/non-Hispanic	91% (878)
% others	9% (91)
*Mild cognitive impairment*	
% yes	16% (154)
% no	84% (815)

Non-residualized scores reported for biomarkers

^*^Six missing (*N* = 963)

^+^Thirty-three missing (*N* = 936)

### Blood collection and processing

For ADNI participants, plasma and serum samples were collected under fasting conditions. NfL was measured from plasma samples, and creatinine was measured from serum samples. Fasting began overnight (approximately 8 h) before collection in the morning. All plasma samples were processed per ADNI laboratory standard operating procedures (http://adni.loni.ucla.edu). Plasma NfL was measured with an ultrasensitive single-molecule array (Simoa) platform using a home brew kit (Simoa Homebrew Assay Development Kit; Quanterix Corporation), as described previously [19]. The assay uses a combination of monoclonal antibodies and purified bovine NfL as a calibrator. The standard exclusion criteria included hemolysis and a coefficient of variation in plasma concentrations >.25. For serum samples, they were processed by Covance Laboratory kits using isotope dilution mass spectrometry as part of the clinical lab data generated for ADNI.

For VETSA participants, plasma and serum samples were collected under fasting conditions. NfL was measured from plasma samples, and creatinine was measured from serum samples. Fasting began by 9:00 PM the night before testing, and samples were acquired the following morning between 8:00 AM and 8:15 AM. For plasma samples, NfL was assayed on a single-plex plate using the ultra-sensitive Simoa technology platform HD-1 (Simoa NFL Advantage Kit; Quanterix Corporation) by the USC Alzheimer’s Therapeutic Research Institute Biomarker Core (PI: Dr. Robert Rissman) [20], and all assays were performed according to the manufacturer’s instructions. The standard exclusion criteria included hemolysis and a coefficient of variation in plasma concentrations >.20. For serum samples, they were processed by the Quest Diagnostic using spectrophotometry as part of the blood chemistry data generated for VETSA.

### Cerebrospinal fluid collection and processing

For ADNI participants, cerebrospinal fluid (CSF) was sampled by lumbar puncture per ADNI laboratory standard operating procedures (http://adni.loni.ucla.edu) on the same day of the blood draw for plasma NfL. The CSF NfL concentration was measured using a commercially available enzyme-linked immunosorbent assay (ELISA) following the protocol provided by the manufacturer (NF-light; Uman Diagnostics) as described previously [21]. Intra-assay coefficients of variation were <10%. Additional procedures for CSF collection, processing, and storage procedures have been described previously [22].

### MRI acquisition and processing

For ADNI, acquisition parameters have been described in detail previously [23]. Briefly, T1-weighted images for ADNI were collected from 3.0-T scanners, using protocols optimized for each scanner system. Detailed acquisition parameters can be found at https://adni.loni.usc.edu/methods/documents/mri-protocols/. Total cortical, medial temporal lobe, and hippocampal volume measures (UCSFFSX51_ADNI1_3T_02_01_16.csv) were derived using the FreeSurfer 5.1 (surfer.nmr.mgh.harvard.edu) software package. For more detailed information on preprocessing and quality control, please see the full UCSF FreeSurfer Overview and QC Guide and UCSF FreeSurfer Methods in the ADNI database.

For VETSA, acquisition parameters have been described in detail previously [24, 25]. Briefly, T1-weighted images (sagittal 3D fast spoiled gradient echo (FSPGR), TE = 3.164 ms, TR = 8.084 ms) were acquired on two General Electric (GE) Discovery MR750 3.0T scanners (GE Healthcare, Waukesha, WI, USA) at UCSD with an eight-channel phased array head coil. The structural MR images were processed as described previously [24–26]. Total cortical, medial temporal lobe, and hippocampal volume measures were derived using the FreeSurfer 6.0 (surfer.nmr.mgh.harvard.edu) software package. Preprocessing included correction of distortion due to gradient nonlinearity, image intensity normalization, and rigid registration into standard orientation with 1 mm isotropic voxel size. All images required some form of manual intervention to ensure the correct classification of the white matter and pial surfaces, either with normalization control points or manual editing of white matter or brain masks. Problematic cortical reconstructions were reviewed by consensus with 3 neuroimaging analysts.

### Statistical analyses

Statistical analyses were performed using R version 4.1.2. Correlational analyses for study 1 and twin analyses for study 2 were performed using the raw data application of the maximum likelihood-based structural equation modeling software OpenMx version 2.19.8 [27]. For ADNI participants, serum creatinine, plasma NfL, and CSF NfL were adjusted for (1) age, (2) race/ethnicity (White/non-Hispanic or others), and (3) body mass index, using the *umx_residualize()* function [24]. Information on the storage time of blood samples was not available for ADNI data. The testing site was not adjusted given the blood collection protocols were standardized across sites. For VETSA participants, measures of serum creatinine and plasma NfL were residualized using the *umx_residualize()* function [28] to account for (1) age, (2) race/ethnicity (White/non-Hispanic or others), (3) body mass index, and (4) whether or not twin pairs were assessed on the same day. Plasma NfL was additionally adjusted for testing site and sample storage time. Residualized scores of all measures from both datasets were then log-transformed to improve their distribution properties. For each measure, data points that were more than three times the interquartile range above the third quartile were excluded from further analyses. For the ADNI sample, 2 plasma NfL data points, 2 CSF NfL data points, and 1 serum creatinine data point were excluded. For the VETSA sample, 3 plasma NfL data points were excluded.

Brain volumetric measures in both samples were residualized to account for (1) age, (2) race/ethnicity (White/non-Hispanic or others), and (3) intracranial volume. Sex was adjusted for in the ADNI sample. Scanner differences were adjusted for the VETSA sample, but not the ADNI sample as the data were standardized across collection sites.

#### Biometrical twin analyses

**Fig. 1:**
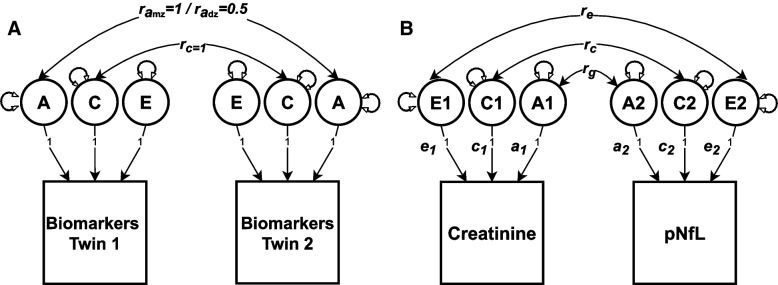
Biometrical twin modeling. **A** Univariate biometrical ACE model. A = additive genetic influences, C = common/shared environmental influences, E = unique environmental influences, r_amz_ = correlation within MZ twin pairs, r_adz_ = correlation within DZ twin pairs, r_c_ = correlation within MZ and DZ twin pairs. **B** Bivariate correlated factor model with creatinine and plasma NfL. For simplicity, only one twin is shown for the bivariate model. Genetic (r_g_), common environmental (r_c_), and unique environmental (r_e_) correlations between the two biomarkers were estimated

To determine the relative contributions of genetic and environmental influences on the variance in creatinine and plasma NfL, we fitted univariate and bivariate biometrical models to the data. In univariate twin analysis, the variance in a phenotype (e.g., biomarker) is decomposed into additive genetic (*A*) influences, common or shared environmental (*C*) influences (i.e., environmental factors that make members of a twin pair similar to one another), and non-shared or unique environmental (*E*) influences (i.e., environmental factors that make members of a twin pair different from one another, including measurement error) (Fig. 1A) [29, 30]. This approach is also referred to as the biometrical “ACE” model.

The decomposition is achieved by exploiting the expected genetic and environmental correlations between MZ and DZ twin pairs. MZ twin pairs are genetically identical, whereas DZ twin pairs share, on average, half of their genes. Therefore, the MZ and DZ twin pair correlations for the additive genetic effects are fixed to rA=1.0 and rA=0.5, respectively. The modeling assumes that the sharing of environmental effects (*C*) is equal in MZ and DZ twin pairs (rC=1.0), while non-shared environmental effects (*E*) are, by definition, uncorrelated within twin pairs and include measurement errors. The biometrical “ACE” model based on twins reared together also assumes no genotype by environmental interactions or correlations and non-random mating.

In bivariate twin analyses, the univariate model is extended to estimate the *A*, *C*, and *E* within each biomarker and the magnitude and significance of shared genetic and environmental influences between the two biomarkers. Specifically, we fitted a bivariate ACE “correlated factors” model [31] to the data (Fig. 1B) to estimate the genetic and environmental correlations that represent the degree to which genetic and environmental influences are shared between biomarkers.

For both univariate and bivariate analyses, we determined the most likely sources of variance by fitting a series of competing sub-models (AE, CE, and *E*) in which the (i) *C*, (ii) *A*, and (iii) *C* and *A* influences were dropped and fixed to 0. The sub-models were then compared to the full ACE model using the likelihood ratio chi-square tests. The best-fitting model was determined based on the optimal balance of complexity and explanatory power by using the Akaike Information Criterion (AIC) [32] and likelihood ratio chi-square tests.

## Results

### Associations of biomarkers with covariates

For ADNI participants, we found that age at assessment (*β*=0.99, *t*=4.88, *p*<0.001) and body mass index (*β*=− 0.95, *t*=− 2.96, *p*=0.003) were the two covariates showing significant associations with plasma NfL. For CSF NfL, age at assessment was a significant covariate (*β*=28.20, *t*=3.20, *p*=0.002). For serum creatinine, age at assessment (*β*=0.01, *t*=3.40, *p*<0.001) and body mass index (*β*=0.01, *t*=3.41, *p*<0.001) were significant covariates. Race/ethnicity was not associated with individual differences in any of the biomarkers.

For VETSA participants, we found that age at assessment (*β*=0.28, *t*=3.19, *p*=0.001) and body mass index (*β*=− 0.12, *t*=− 2.80, *p*=0.005) were the two covariates showing significant associations with plasma NfL. For serum creatinine, only body mass index was a significant covariate (*β*=0.003, *t*=2.36, *p*=0.018). Individuals in the White/non-Hispanic group had lower serum creatinine than those in the other group (*β*=− 0.07, *t*=− 3.11, *p*=0.002). Whether or not twins were assessed on the same day was not associated with individual differences in any of the biomarkers. Likewise, testing sites and sample storage time were not associated with plasma NfL.

### Study 1: Associations among plasma NfL, CSF NfL, and serum creatinine

**Fig. 2:**
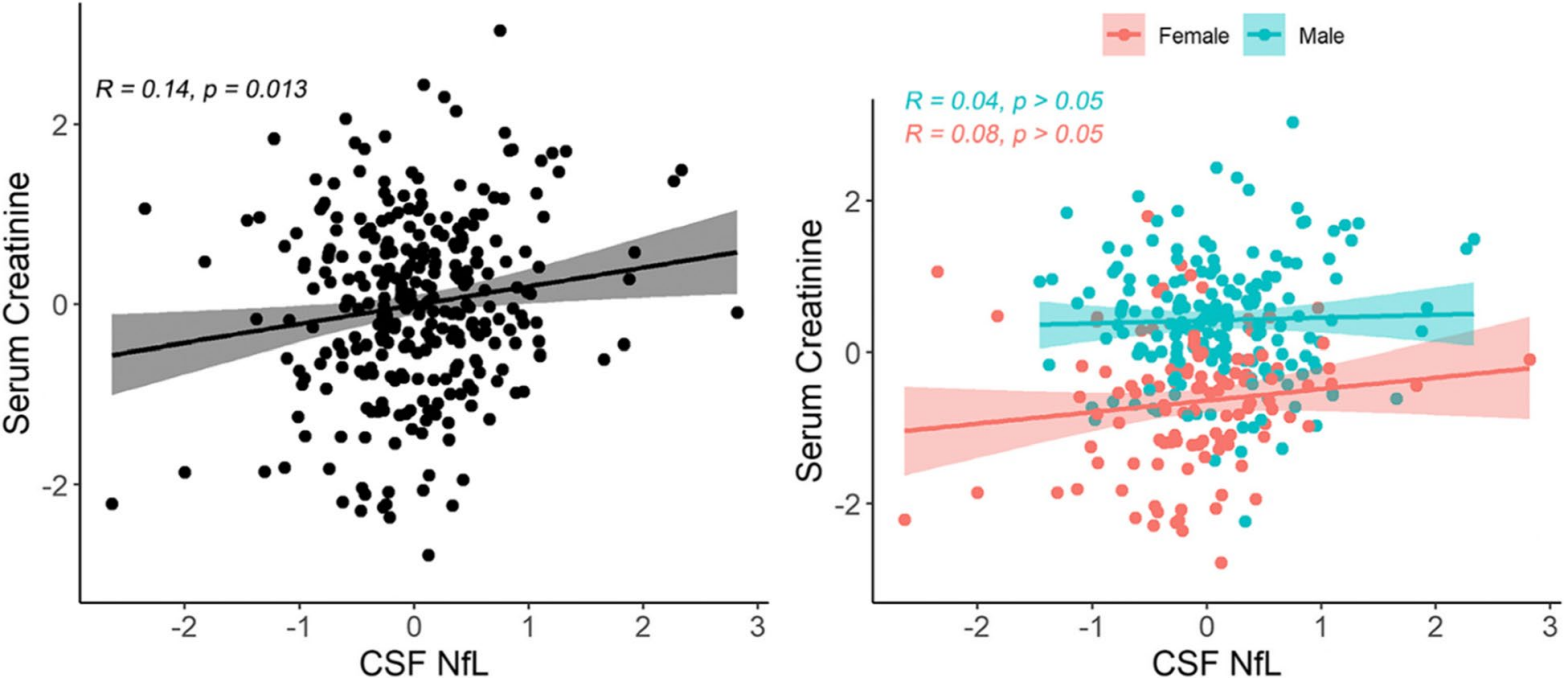
Scatterplots of CSF NfL and serum creatinine correlation in the ADNI sample. The left panel is the full sample, and the right is separated by sex group. Both biomarkers were log-transformed

Consistent with prior work [16], we detected a correlation between plasma NfL and CSF NfL with similar magnitudes in the ADNI dataset (full sample: *r*=0.53, 95% CI: 0.44–0.61, *p*<0.001; male: *r*=0.56, 95% CI: 0.45–0.66, *p*<0.001; female: *r*=0.54, 95% CI: 0.41–0.66, *p*<0.001). Likewise, plasma NfL and creatinine were significantly correlated (*r*=0.26, 95% CI: 0.17–0.35, *p*<0.001), but the correlations appeared to be numerically but not significantly stronger in males (*r*=0.39, 95% CI: 0.27–0.49, *p*<0.001) than in females (*r*=0.22, 95% CI: 0.06–0.37, *p*=0.006). Creatinine and CSF NfL were modestly correlated in the full sample (*r*=0.14, 95% CI: 0.03–0.24, *p*=0.013) but no longer correlated after controlling for sex (*r*=0.06, 95% CI: − 0.05–0.17, *p*>0.05) or separating the sample by sex (male: *r*=0.04, 95% CI: − 0.10–0.17, *p*>0.05; female: *r*=0.08, 95% CI: − 0.10–0.26, *p*>0.05) (Fig. 2).

Next, we examined whether statistically controlling for the effect of creatinine on plasma NfL alters its association with CSF NfL. If plasma NfL level is affected by renal function and does not solely reflect neurodegeneration, then adjusting plasma NfL for creatinine could significantly change its association with CSF NfL. After adjustment, there was a negligible change (±0.02) in the magnitude of correlations between plasma NfL and CSF NfL (full sample: *r*=0.51, 95% CI: 0.41–0.59, *p*<0.001; male: *r*=0.58, 95% CI: 0.47–0.67, *p*<0.001; female: *r*=0.56, 95% CI: 0.42–0.67, *p*<0.001). Analyses on brain volumetric measures revealed the same patterns, such that plasma NfL’s correlations with these measures differed little (±0.03) after adjusting for creatinine in the ADNI sample (Table 3 top panel).

**Table 3:** Correlations of plasma NfL with brain structural measures in the ADNI and VETSA samples

ADNI—brain structures	Plasma NfL	*p* value	Plasma NfL adjusted	*p* value
Hippocampal volume	− 0.21 [− 0.31, − 0.11]	< 0.001	− 0.18 [− 0.28, − 0.07]	< 0.001
Medial temporal lobe volume	− 0.12 [− 0.23, − 0.01]	0.033	− 0.11 [− 0.23, − 0.00]	0.047
Whole brain volume	− 0.22 [− 0.32, − 0.12]	< 0.001	− 0.21 [− 0.30, − 0.11]	< 0.001
**VETSA—brain structures**	**Plasma NfL**	***p*** **value**	**Plasma NfL adjusted**	***p* value**
Hippocampal volume	− 0.04 [− 0.15, 0.06]	> 0.05	− 0.03 [− 0.14, 0.07]	> 0.05
Medial temporal lobe volume	0.07 [− 0.03, 0.17]	> 0.05	0.05 [− 0.05, 0.15]	> 0.05
Whole brain volume	0.01 [− 0.09, 0.11]	> 0.05	0.02 [− 0.08, 0.11]	> 0.05

Correlations of plasma NfL are shown with and without adjusting for creatinine. Structural measures were adjusted for age, sex when applicable, and intracranial volume

### Study 2: Replications and twin analyses

We replicated the same analyses examining the associations between plasma NfL and brain volumetric measures before and after adjusting for creatinine levels in the VETSA sample. Not surprisingly, these associations did not meaningfully change (±0.02) (Table 3 bottom panel). Similarly, consistent with prior findings [7, 8, 13] and results in the ADNI sample, the phenotypic correlation between creatinine and plasma NfL was significant (*r*=0.38, 95% CI: 0.32–0.43, *p*<0.001). The DZ twin pair correlations (creatinine: *r*=0.31, 95% CI: 0.15–0.46, *p*<0.001; plasma NfL: *r*=0.23, 95% CI: 0.06–0.38, *p*=0.008) were less than or equal to one-half the size of MZ twin pair correlations (creatinine: *r*=0.64, 95% CI: 0.56–0.71, *p*<0.001; plasma NfL: *r*=0.57, 95% CI: 0.48–0.65, *p*<0.001), which is consistent with additive genetic influences.

### Tests of mean and variance homogeneity

Prior to the twin modeling, we tested the assumption of mean and variance homogeneity within and across the MZ and DZ twin groups for each biomarker using the residualized data. We compared the constrained models that equated mean and variance between and across zygosity to a fully saturated model that perfectly reproduced all mean and variance-covariance information for each biomarker.

We conducted the chi-square tests to compare the constrained models to the fully saturated model. A significant change in chi-square (*p*=0.05, Bonferroni corrected *p*=0.01) would suggest that there is a significant difference between the models (i.e., actual mean and variance vs. constrained mean and variance), indicating that the assumption of mean and variance homogeneity is violated. As shown in Table 4, constraining the means and variances to be equal within twin pairs and across zygosity resulted in a significant change in chi-square for creatinine (*p*<0.01), but not for NfL (*p*>0.01). This was likely attributable to the small numbers of complete and incomplete twin pairs within each zygosity group. Notwithstanding this limitation, all subsequent analyses proceeded under the assumption of mean variance homogeneity for each biomarker.

**Table 4:** Mean and variance homogeneity testing within and across zygosity for serum creatinine and plasma NfL

**Serum creatinine**	**ep**	**− 2LL**	**df**	**AIC**	**Δ**− **2LL**	**Δdf**	***p***
Saturated model	10	2518.76	926	2538.76			
Mean within zygosity	8	2526.25	928	2542.25	7.49	2	0.024
Mean across zygosity	7	2526.50	929	2540.50	7.75	3	0.052
Variance within zygosity	5	2531.85	931	2541.85	13.09	5	0.023
Variance across zygosity	4	2536.65	932	2544.65	17.90	6	0.006
**Plasma NfL**	**ep**	**− 2LL**	**df**	**AIC**	**Δ− 2LL**	**Δdf**	***p***
Saturated model	10	2619.35	950	2639.35			
Mean within zygosity	8	2620.83	952	2636.83	1.49	2	0.475
Mean across zygosity	7	2624.17	953	2638.17	4.83	3	0.185
Variance within zygosity	5	2630.01	955	2640.01	10.61	5	0.059
Variance across zygosity	4	2633.55	956	2641.55	14.21	6	0.027

Model names specify which parameters were tested for equality

*ep* number of estimated parameters, *− 2LL* − 2 × log-likelihood, *Δ− 2LL* change in − 2 × log-likelihood, *Δdf* change in degrees of freedom, *AIC* Akaike Information Criteria

We also tested the assumption of mean and variance homogeneity by comparing the bivariate correlated factor model to a fully saturated model that reproduces perfectly all mean and variance-covariance information for the observed variables. Although the bivariate ACE model resulted in a significant difference in fit compared to the fully saturated model, all other fit indices (i.e., lower AIC, CFI>0.9, RMSEA<0.05) suggested that the bivariate model fits the data well.

### Estimation of genetic and environmental influences

In both univariate and bivariate twin analyses, the AE models provided the best fitting to the data as judged by the lowest AIC values and non-significant changes in − 2 log-likelihood (-− 2LL) when compared to the ACE models (Table 5). Univariate and bivariate analyses yielded essentially identical estimates of A and E for both biomarkers.

**Table 5:** Bivariate and univariate model fitting comparisons under the competing ACE, AE, CE, and E models

Bivariate	Model	ep	− 2LL	df	Δ− 2LL	Δdf	*p*	AIC
Correlated factors	ACE	11	4980.17	1885				5002.17
	**AE**	**8**	**4981.28**	**1888**	**1.11**	**3**	**0.7374**	**4997.28**
	CE	8	5001.96	1888	21.79	3	< 0.001	5017.96
	E	5	5173.55	1891	193.38	6	< 0.001	5183.55
**Univariate**	**Model**	**ep**	**− 2LL**	**df**	**Δ− 2LL**	**Δdf**	***p***	**AIC**
Creatinine	ACE	4	2536.65	932				2544.65
	**AE**	**3**	**2537.12**	**933**	**0.47**	**1**	**0.4945**	**2543.12**
	CE	3	2546.23	933	9.58	1	0.0020	2552.23
	E	2	2655.25	934	118.60	2	< 0.001	2659.25
Plasma NfL	ACE	4	2633.55	956				2641.55
	**AE**	**3**	**2633.62**	**957**	**0.07**	**1**	**0.7952**	**2639.62**
	CE	3	2645.60	957	12.05	1	< 0.001	2651.60
	E	2	2723.36	958	89.81	2	< 0.001	2723.36

Best fitting model is in bold font

*A* additive genetic, *C* common or shared environment, *E* non-shared environment, *ep* number of estimated parameters, *− 2LL* − 2 × log-likelihood, *Δ− 2LL* change in − 2 × log-likelihood, *Δdf* change in degrees of freedom, *AIC* Akaike Information Criteria

Here, we report the bivariate results, as they provide more precise estimates than univariate analyses. Additive genetic influences accounted for 60% (95% CI: 52–67%) of the variance in creatinine and 54% (95% CI: 45–62%) of the variance in plasma NfL, and there was a significant genetic correlation between them (*r*_*g*_=0.46, 95% CI: 0.34–0.57, *p*<0.001). There was also a smaller but significant non-shared environmental correlation between the biomarkers (*r*_*e*_=0.27, 95% CI: 0.15–0.38, *p*<0.001).

## Discussion

Considering comorbidities in research and clinical practice using blood-based biomarkers of neurodegenerative disorders is critical for establishing generalizable cutoffs under different contexts of use and avoiding potential misdiagnosis in real-world settings [10, 11, 15]. In two different study samples, we examined how renal function impacts plasma NfL levels and their accuracy in reflecting neurodegeneration. We showed that adjusting for the effect of serum creatinine levels (i.e., renal function) on plasma NfL levels did not meaningfully change plasma NfL’s association with CSF NfL or brain volumetric measures that index neurodegeneration. To tease apart the underlying mechanisms of the NfL-renal function association, we leveraged twin modeling to show that not only were plasma NfL and creatinine both heritable (*h*^2^=0.54 and 0.60, respectively), but also their association was accounted for to a moderate degree by shared genetic factors, with the remaining variance accounted for by unique environmental factors.

A previous study showed that among all known factors associated with blood-based NfL, body mass index was the only significant factor associated with blood-based NfL levels in individuals less than 60 years old. In those who are greater than 60 years old, however, blood-based measures of renal function (i.e., creatinine, eGFR) became significantly correlated with blood-based NfL levels [8]. Indeed, studies that showed associations were all in samples above the age of 60 [7, 8, 13], during which diminished renal function becomes more common and/or may be more difficult to compensate for. Our observed phenotypic associations between creatinine and plasma NfL in the ADNI sample with a mean age of 75 years and in the VETSA sample with a mean age of 67 years are consistent with those findings. As such, one prominent hypothesis is that renal dysfunction in old age, albeit not reaching the end or severe stages of chronic kidney disease, may reduce the peripheral clearance of NfL in the blood [7, 10, 13]. In fact, it has been suggested that smaller fragments of NfL resulting from neurodegeneration or metabolism of NfL could be filtered at the glomerular level in the kidney [13]. Thus, renal dysfunction in old age may lead to the accumulation of these fragments, which may then be recognized by the NfL antibody in blood-based assays [13].

Using biometrical twin modeling, our results from the VETSA sample offered unique insights into the association between plasma NfL and serum creatinine by showing the extent of overlapping genetic influences. These findings may lay the groundwork for future genome-wide association studies (GWAS), which would be able to identify what the shared genes are and provide more information on potential cellular and molecular pathways involving both biomarkers. If peripheral clearance through the kidney [33–35] is indeed what contributes to the association between blood-based measures of renal function and NfL, then the detection of genes related to renal clearance in GWAS would provide concrete evidence in support of this hypothesis. Nonetheless, more large-scale studies are needed to validate our findings and empirically test the clearance hypothesis at molecular and cellular levels.

**Fig. 3:**
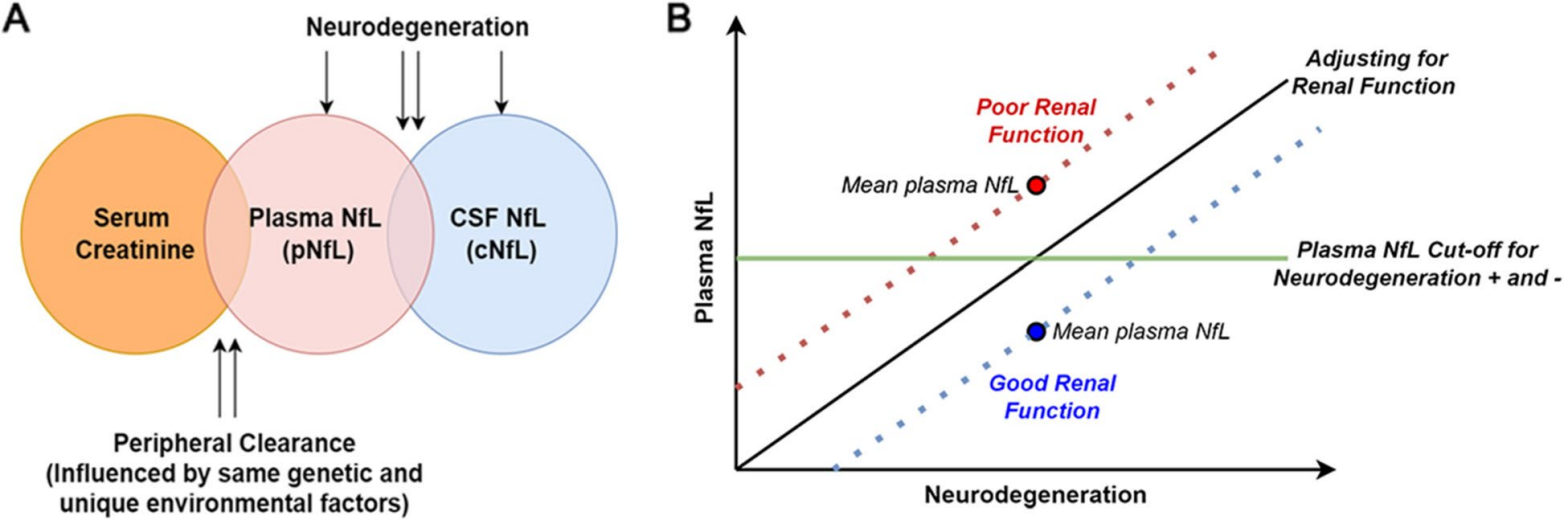
Conceptual diagram of plasma NfL, CSF NfL, and serum creatinine. **A** Venn diagram of the associations among plasma NfL, CSF NfL, and serum creatinine. Shared variance between plasma NfL and serum creatinine is independent of the shared variance between plasma NfL and CSF NfL. **B** Conceptual plot of plasma NfL indexing the same degree of neurodegeneration in groups with good or poor renal function and in groups after controlling for renal function. Renal function affects the level of plasma NfL and therefore how it performs as a stand-alone measure (e.g., when compared to a threshold for abnormality as shown in the green line). However, adjusting for renal function does not alter the slope of association between plasma NfL and another measure of neurodegeneration

For researchers and clinicians, one major concern regarding the association between blood-based measures of renal function (in this case serum creatinine) and NfL is that reduced clearance of blood NfL fragments may increase the level of plasma NfL, thereby reflecting an inaccurate and potentially inflated estimate of neurodegeneration [7, 8, 13]. If poor renal function weakens the relationship between plasma NfL and other measures of neurodegeneration, then creatinine should always be controlled for when using plasma NfL as a biomarker of neurodegeneration. Alternatively, if poor renal function is associated with increased neurodegeneration, the association between plasma NfL and creatinine could be driven by a common process. In this case, creatinine should not be controlled for when measuring plasma NfL. The associations of plasma NfL with CSF NfL in the ADNI sample and with brain volumetric measures in both samples were unaffected by creatinine levels. Thus, we speculate that the portion of the variance in plasma NfL that is related to neurodegeneration is independent from the variance related to renal clearance (Fig. 3A). Consequently, covarying for creatinine levels in plasma NfL would not meaningfully alter the strength and direction of its associations with other markers of neurodegeneration. We thus recommend not adjusting for creatinine levels in such analyses.

However, renal function and potentially other health conditions (see [5] for an example approach) should be accounted for when plasma NfL is compared to a cutoff to classify individuals as positive or negative for neurodegeneration [13, 14, 36]. This is because the level of plasma NfL is determined by the full variance of plasma NfL, including that shared with renal function (i.e., the entire plasma NfL circle in Fig. 3A). As shown in extant literature [10, 11], individuals or groups with poor renal function tend to have higher plasma NfL than those with good renal function. Thus, the 2 groups based on renal function may have different plasma NfL levels, but may not differ on neurodegeneration (Fig. 3B). Thus, for this purpose, adjusting for renal function may be necessary to ensure comparable estimates and categorization of neurodegeneration across groups or among individuals with varying levels of renal function. Relatedly, when comparing the associations of plasma NfL with other measures among the groups with a significant mean difference in renal function, investigators may run into Simpson’s paradox [37] (i.e., when an association in the full sample disappears or reverses when it is divided into subgroups) and would need to adjust for renal function to reveal the more accurate relationship.

Finally, our study has some limitations. First, the twin sample only has male participants. Thus, we cannot determine if there are sex differences in the genetic and environmental influences on these biomarkers. However, our results from the ADNI sample and prior reports all detected a phenotypic association between blood-based measures of NfL and renal function in both sexes with comparable magnitudes, suggesting no sex difference in the association [7–9, 13]. Second, because both samples are largely White, non-Hispanic, our results may not be generalizable to other racial/ethnic groups. Third, different collection and processing procedures of the biomarkers between the two studies may lead to potential differences in biomarker levels. For example, storage time differences between the two studies may lead to potential differences in biomarker levels. Serum creatinine was processed differently between the two datasets (spectrophotometry vs. isotope dilution mass spectrometry) at two different laboratories. Additionally, plasma NfL was processed using different kits (Simoa Homebrew Assay Development Kit vs. Simoa NFL Advantage Kit). Fourth, different ages and proportions of MCI cases (see Tables 1 and 2) between the two studies may lead to differences in mean biomarker levels. However, despite those differences, results from both studies on the association between plasma NfL and creatinine were consistent, strongly supporting the generalizability of our findings. Fifth, our samples focused on older adults with mean ages of 67 and 75, which may not generalize to younger age groups. For individuals that are less than 60–65 years of age, the diagnostic cutoffs for plasma NfL levels [14, 36] and the association between plasma NfL and creatinine [8] may be different from those in the older age groups. Sixth, our study only examined one measure of renal function (i.e., serum creatinine), but other indices of renal function such as cystatin C, which has been reported to have high sensitivity for detecting renal impairment [13], could also be examined in relation to plasma NfL. We expect similar genetic and environmental influences would be detected in other blood-based measures of renal function, as similar phenotypic associations have been observed across different measures of renal function [13].

## Conclusions

Taken together, the present study is the first to investigate whether adjusting for renal function alters plasma NfL’s accuracy in indexing neurodegeneration and examine the genetic and environmental determinants of the associations between blood-based measures of NfL and renal function. In two different study samples, we showed that adjusting for creatine levels is unlikely to affect correlations between plasma NfL and other measures of neurodegeneration, but it is likely to be important if plasma NfL is compared to a cutoff for classifying neurodegeneration-positive versus neurodegeneration-negative individuals. Future work would benefit from comparing the associations of NfL obtained from the blood and CSF with different biomarkers of renal function in both younger and older age groups to better understand the nature of their relationships.

## Data Availability

The Alzheimer’s Disease Neuroimaging Initiative (ADNI) dataset is publicly available on its database (http://adni.loni.usc.edu). The Vietnam Era Twin Study of Aging dataset is publicly available to qualified researchers, with restrictions. Information regarding data access can be found at http://www.vetsatwins.org/for-researchers/.

## Abbreviations

AD: Alzheimer’s disease
AIC: Akaike Information Criterion
CFI: Comparative fit index
CSF: Cerebrospinal fluid
DZ: Dizygotic
eGFR: Estimated glomerular filtration rate
GWAS: Genome-wide association studies
MCI: Mild cognitive impairment
MZ: Monozygotic
NfL: Neurofilament light chain
RMSEA: Root mean square error of approximation
